# Treating symptoms or assisting human development: Can different environmental conditions affect personal development for patients with severe mental illness? A qualitative study

**DOI:** 10.1186/s13033-016-0041-2

**Published:** 2016-02-18

**Authors:** Arnhild Lauveng, Sidsel Tveiten, Tor-Johan Ekeland, Ruud Torleif

**Affiliations:** Division Mental Health Services, Akershus University Hospital, Lørenskog, Norway; Institute for Clinical Medicine Oslo, University of Oslo, Oslo, Norway; Department of Health, Faculty of Health Sciences, Nutrition and Management, Oslo and Akershus University College of Applied Sciences, Oslo, Norway; Volda University College, Volda, Norway

## Abstract

**Background:**

Recent research suggests that a basic anomaly in self-experience may be a core factor in patients with severe mental illnesses. Given the importance of sense of self, the traditional treatment of symptoms might not be the most effective for these groups of patients. This qualitative study examines how differences in social environmental conditions, organized as education or treatment, might affect personal development in patients with severe mental illness.

**Methods:**

A qualitative hermeneutical design was used. Data were collected through qualitative interviews. Informants included 14 patients in psychiatric treatment and 15 students at schools for adults with mental illness. Most informants were interviewed on two occasions, 6–8 months apart, totaling 47 interviews. All participants had been diagnosed with severe mental illness with pronounced impact on daily functioning (most often psychoses or personality disorders) for a minimum of 2 years.

**Results:**

Findings and interpretations showed that the students experienced a supportive environment focused mostly on education. They described personal and enduring development in areas such as capacity for relationships, regulation of symptoms, subjective well-being, and integration in society. The patients experienced an environment focused more on treatment of their illness and less on personal development and interests. They described little development, much loneliness, a poor quality of life, an objectifying attitude of themselves and others, and hopelessness.

**Conclusions:**

Even if more research is needed, findings indicate that for this group of patients, problems may be closely related to identity development. Therefore, instead of solemnly focusing on specific symptoms, it might be more effective to support patients’ personal and social development by offering intensive and lasting social environmental conditions. This includes stable and mutual relationships, intrinsically motivated activities, and an environment that supports personal choices, acceptance, and development.

## Background

Personal development and sense of self seems to be important concepts in understanding severe mental illnesses. Recent research on the association between sense of self and schizophrenia suggests that a basic anomaly in self-experience may be a core factor in the syndrome [1, 2]. Other research on self-experience and personality disorders, especially borderline personality disorders [3] focuses more on anomalies in the narrative sense of self.

A large body of research also suggests a strong association between relational trauma in childhood and severe mental illness [4, 5]. Most developmental theories state that relationships are crucial for identity development and that identity is created in interactions with others [6–8]. Through language and interaction, the individual recreates the same attitude toward himself as society has toward him; he will think of and treat himself according to how others speak to and treat him [6], and other theories echo this [7–9]. Children who experience developmental trauma may have problems with relationships and sense of self. This “wrong learning” is addressed in various ways in psychiatric treatment, with biological and behavioral methods as well as relational, group, and milieu therapy. A common feature of these therapies is that patients’ symptoms are treated by experts, and patients and professionals do not experience mutual interaction in daily life [10].

However, mutual interaction in daily life is the normal way for children to develop a sense of self and may be especially important for helping traumatized children overcome difficulties [11]. Therefore, a potential alternative or complement to medical intervention may be to help adult patients, regardless of symptoms and disease, experience opportunities for development similar to the ways children do. For children, this development does not usually take place within the health care system but in families, schools, and communities as continuous interactions with others that serve to confirm their identity and meet developmental challenges. Sweden’s Family Care Foundation demonstrates positive results working with children, teenagers, and adults in foster homes where daily interaction with the foster family is combined with psychotherapy to assist personal development in patients with severe mental illness [12]. In other contexts, focusing on daily life and interactions seems to be important over time for the development of patients with severe and long-term illnesses, a view supported by statements from patients themselves [10, 13].

This body of research suggests that, for some patients with severe, long-lasting problems related to their experience of sense of self and childhood developmental trauma, the traditional model of treating symptoms might be less effective because the symptoms may be manifestations of deeper problems regarding identity and sense of self. Therefore, it is relevant to explore how agencies other than the health care system assist adults in the process of developing a more secure sense of self. Furthermore, it is useful to explore the potential impacts such agencies have on patients’ quality of life, ability to function in daily life, and level of symptoms.

In Denmark, there are schools for adults with different types of challenges including mental disorders [14]. Most students attend school 3–5 days a week. Together with their supervisors, they develop their own schedules from a wide range of subjects such as philosophy, social studies, language, arts and crafts, sports, outdoor activities, cooking, mathematics, literature, science, and more. Younger students have agreements with the municipality that limit the duration of their attendance; older students with disability pensions may attend for as long as they wish. Such schools offer not only activities that differ from most traditional psychiatric treatments but also a different *framing* that focuses on education and development instead of illness and treatment. In an earlier paper [15], we discussed how students at these schools and patients in psychiatric treatment describe their life situations quite differently. In this paper, we will examine whether differences related to development can also be found.

## Aim and research questions

The aim of this study was to identify and examine possible differences in descriptions of self and of personal development related to a being in an educational environment versus being in a treatment environment with focus on symptoms of mental illness.

The following three research questions are addressed: (1) How do patients in treatment and students in education describe the development of their relationships and interactions? (2) How do patients in treatment and students in education describe themselves, their motivations, and their actions? (3) How do patients in treatment and students in education describe their own personal development?

## Methods

A qualitative design with individual open interviews to gather the data was used. The freely told story of the informant as a response to open questions was listened to repeatedly and interpreted based on hermeneutical principles (whole-parts, pre-understanding-understanding, context and language) in the analysis.

### Informants and data collection

A total of *14 patients* were interviewed from two different community mental health centers in Norway; nine patients were interviewed twice (6–12 months apart) and five were interviewed once, totaling 23 interviews. Each interview had about 60–80 min’ average duration. Initial interviews were conducted when the patients were admitted to a short-term open ward. At the time of the second interview, most patients were living at home after discharge from the ward.

A total of *15 students* participated in the study. Since such schools do not exist in Norway, students were recruited at two different Danish schools for adults with mental illnesses. These schools have much in common with a tradition in Denmark and Norway of ≪boarding high schools≫ (non-degree granting 1 year colleges) offering subjects and training for personal development more than academic education, and without exams. They are not based on patient identities and thus differ from recovery colleges in the UK. All interviews were conducted at the schools; six students were interviewed once and nine students were interviewed twice (6–12 months apart), for a total of 24 interviews.

All patients, and nine of the students, were asked and agreed to take part also in a second interview 6 or more months later, mainly because the patients were inpatients at the first interview and would be expected to be at home and in a more ordinary living situation at the second interview. However, three patients did not take part. One did not meet in spite of three agreed times for interview, one was in terminal phase of cancer and one had moved and was not able to be found. Two of the second interviews of patients were used to a limited extent in analyses, as they were not tape recorded because one refused recording and the other only accepted a telephone interview. One student was only interviewed once as she was not available during the first period of interviews at the school but wanted strongly to take part in the second period. There are few such schools in Denmark, and it took so long to find the second school that the five students from this school were included very late in the study. For practical reasons it was therefore only possible with one interview. It was also considered less important with two interviews for the students, since they were at school, with similar living conditions, at the time for both interviews.

*Inclusion criteria* for both groups were that informants had been diagnosed with a severe mental illness with substantial impairment and duration of at least 2 years. Informants needed to speak Norwegian or Danish and be able to participate in interviews. At the wards, nurses invited patients they considered to meet the criteria to participate. At the schools, teachers asked some students directly, and I attended a morning gathering and provided information about the study. Lists were available to sign for any student meeting the criteria and wishing to participate.

For *informants’background details,* see Table 1. All information is from the informants’ own descriptions. Many had received several different diagnoses, and some were unsure which diagnoses were still considered valid.

**Table 1:** Background of informants

Background information	Patient (N = 14)	Students (N = 15)
Diagnoses^a^		
Anxiety/depression	8	5
Bipolar disorder		1
Schizophrenia, other psychotic disorder	6	7
Personality disorder	4	8
ADHD, learning disorders, autism spectrum disorder	2	3
Dependency (alcohol, drugs)	8	3
Other	1	
Age		
18–29	7	7
30–49	5	5
50 or older	2	3
Gender		
Male	9	7
Female	6	8
Duration of problems		
2–5 years	1	
6–10		
11–20	1	
Since childhood/adolescence	12	15

^a^Many informants reported more than one diagnosis

### Interview

The qualitative interview was inspired by the “Form of Living Interview” [16], which focuses in detail on what a child informant did yesterday. This interview has been in limited use and there is no documentation on assessment of it. It was chosen as an inspiration because it was considered to be a good way to explore the informant’s everyday life and view of themselves. However, we designed our interview to be related to the situation of adult patients and students. The informants were asked to describe a typical day and week, and then asked for their personal interpretation of the situation. When they mentioned changes, they were asked to elaborate. Follow-up questions was designed to explore whether the day or week was representative, and to explore the informants’ own comments and reflections about what they told. To acquire information about their perceived development, they were asked to draw a curve describing “ups and downs” throughout their lives. They were also asked to introduce themselves as they would to a new acquaintance and to describe their hopes and plans for the future.

All interviews were audiotaped, and the Norwegian/Danish interviews were transcribed by medical secretaries with Norwegian/Danish as their native language.

### Ethical considerations

The Regional Research Ethics Committee (REK) and the Privacy Ombudsman at Akershus University Hospital approved the study. The Danish Ethical Committee for Research informed us that their approval was not required. Participation was voluntary and had no effect on schooling or treatment. All informants received oral and written information about the study and signed a statement of informed consent. To ensure confidentiality, age and gender are not provided with quotes.

### Qualitative data analysis

Interviews were analyzed inspired by the principles of hermeneutic qualitative content analysis [17, 18] using N-VIVO software. Interviews from students and patients were analyzed separately. For each group of informants, meaningful units were identified and grouped in sub-categories that were condensed several times before being abstracted as main categories. Main categories regarding described environmental conditions are presented in an earlier paper [15], while categories describing relationships, sense of self, development, and stagnation are presented here. The analysis process was not linear, but followed the hermeneutical principles moving from parts to the whole and vice versa, repeatedly. The first step of the analysis included reading the transcriptions repeatedly in order to obtain a global understanding of the data. Then, transcription of each interview was read in order to search for meaningful units (words, sentences and sections). Then the meaningful units were condensed into sub categories. Furthermore, the sub categories were abstracted to higher level main categories as shown in Table 2. The researchers discussed the meaningful units, the condensed sub categories and the abstractions into main categories as a form of validation.

**Table 2:** Main and sub-categories

Informants	Main categories	Sub-categories	
Patients	Lack of lasting and meaningful relationships	Loneliness	Humans as tools
	Sense of self and personal narrative	Self-presentation and identity as a patient	Contradictions and spectator in own life (including plans and reality)
	Stagnation, discontinuity and contradictions	No or non-specific experience of development	Hopelessness
Students	Relationships	Development of number and quality of friendships	Development of personal relationships
	Sense of self and personal narrative	Self-presentation and self-acceptance	Personal engagement and integrity
	Development	Changes in symptoms and strategies for coping	Personal growth, happiness, and hopes for the future

## Findings and interpretations

In this section, all quotes from patients/students are in *italics.*

## Patients

### Lack of lasting and meaningful relationships

#### Loneliness

Most patients described loneliness, isolation, and anxiety at home: *I am just alone. Yes. Alone. The weekends are long. Sometimes I just suffer through the days.* A few patients described good relationships with family and some friends. Others described having limited or no contact with others except their health care providers. Most patients described their contacts with professional helpers, especially at the wards, as highly important and “lifesaving” and described the professionals as “kind” and “helping.”

At the same time, their relationships with caring professionals were often described as disjointed and limited. Even outpatients who received the most support had appointments that totaled only a few hours weekly. The rest had 1-hour appointments weekly or every 2 weeks, or 10 min a few times a week; the remainder of their time was spent mostly alone and in their apartments. Patients had little influence in the relationship, as the professionals decided how much time the patient needed and how often. If the professional chose to terminate the relationship or made a job change, the patient had to accept the situation. The brief periods of good relationships at the wards alternated with loneliness at home. Most patients described this situation as unvarying, with little or no development over time, or they described being lonelier now than at an earlier time.

### Humans as tools

Patients met each other mainly at the wards and for a limited time. Many patients said they found this interaction important and useful, since they could learn from other patients’ experiences, get their support with similar experiences, or be encouraged if others were sicker than they were. It was striking how many of their descriptions emphasized *usefulness.* Other patients seemed to be described primarily as *tools* for working with their own problems:

*It was strange, because when we came out from the psychologist, I should work on being social at the ward. And so, there was no one there. Such as, now I am ready to work, and there is no one here. However, they showed up later, and then I could start to talk.*

The word “friend” was seldom used, and no one mentioned being liked or being fond of their co-patients or having developed friendships over time.

### Sense of self and personal narrative

#### Self-presentation and identity as a patient

All informants were asked how they would present themselves to a new acquaintance, and for most, this was a difficult question. Most of them concluded that they would just say their name: *I try to avoid myself, in a way. It will be something like …“Hello, I’m (name). What’s your name?”* Some would add that they were ill; none mentioned other topics they would include.

The histories of the patients’ lives, their self-descriptions, and their descriptions of treatment and diagnoses were often interwoven. Many seemed to identify with the role of patient or described a development where the role of patient had become more important over time. Some patients also explained their actions and choices based on their diagnosis, even when other explanations might be possible.

Patients used not only their illness but also medications to explain emotions. All patients spontaneously talked about medicines, and this seemed to be important for them: *Because the medication is functioning now, I feel I can manage.*

### Being a spectator in one’s own life and contradictions

In the interviews with patients, I found many statements indicating a passive, or even objectifying, attitude toward life and themselves. The patients talk about how other people—not themselves—evaluate and describe them:

*When I was admitted, they said that I was very … that my face was grey as ashes and the expression in my eyes … that I was not okay.… But after staying here for eight days, he (the psychologist) suddenly said that it was a delight to see me and that I was much better.*

Questions such as “How did *you* experience this?” led to little new information, and responses were generally a repetition of other people’s evaluations. Patients made many statements indicating that they put aside their own assessments of themselves and their situation—or even let others *replace* themselves, as one patient describes it: *When I am admitted, they do … in a way, the staff takes over the command. Yes. They replace me.*

Most patients said that it was common for appointments to be cancelled or changed, sometimes without prior notice. They first described an agreement, e.g., *“I meet with the psychologist twice a week,”* but upon being asked follow-up questions, it turned out that the reality was different: *You know (the psychologist) has been sick for a week. Then, suddenly, there have been some meetings… So, I have not seen him twice a week, but that has been the intention….*

This lack of consistency and continuity was also seen in many descriptions of their own lives—descriptions that often seemed contradictory: *This group is very helpful. Yes. It does not work for me at all.*

When asked about what he did yesterday, one patient described a day where he was mostly passive: he had an appointment with his therapist in the morning, sat alone in the garden, watched some TV, and otherwise slept. Later, he stated, *At the ward I am working, man is working with himself morning to night. Really, we have no break. It’s like… a lot is going on, you are working with yourself all the time.*

### Stagnation and discontinuity

#### No or non-specific experience of development

Two patients described a positive development, clear plans for the future, and a better everyday life: *Yes. I have worked a lot myself, and I have a very good network that supports me.* These patients also talked about systematic treatment, more networking, and greater personal engagement.

All the other patients described either a worsening of their situation, stagnation, or—at best—a short-term experience of feeling better. Before discharge, they often said that they were “much better,” and they described their plans for when they got home: *I have been a passive member of the gym for two years. Now I will begin training there every day. I will also use the day*-*care center once a week. It will be fine, you know. I hope.* However, at the time of the second interview, this patient had yet to visit the gym or day-care center. Like most patients discussing their plans, he had not acted on them. Many also talked about a rapid relapse and several hospital admissions between the first and second interviews.

### Hopelessness

With few exceptions, most patients described hopelessness and resignation: *I do not have any plans for the future. I just have had enough now.* When asked about how they hoped their lives would be in 6 months, most had no answer: *I do not know. I have completely given up my whole life.*

## Students

### Relationships

#### Development of quantity and quality of friendships

All of the students reported having more friends now, enjoying being with their friends, and being less lonely. Many described the school, teachers, and fellow students as “family,” and some also had more friends outside of school. Many described changes in their insight into and their capacity for relationships: *To attend the school has had consequences. Both my relationship to myself and the world has changed a lot.* They described taking more responsibility for relationships, having more insight into what was going on, and making active choices to establish, maintain, and sometimes limit new and existing relationships.

#### Development of personal relationships

Several students described that during the years they had attended the school, they had made changes in personal relationships. Some had more contact with their natural family and others less; some had married and others had divorced: *The school gave me courage to break out of a destructive marriage. It was not good for me. If I had not attended school, I would have stayed, because I could not manage to break free by myself.* Regardless of the changes, all students described them as important and good for them, and as active choices—not something that just “happened.”

## Sense of self and personal narrative

### Self-presentation and self-acceptance

Students were also asked how they would introduce themselves to a new acquaintance, and most seemed to find this question easy. They said they would mention their names, and most of them would include other topics, like family (*I am a mother*), hobbies (*I love fitness)*, origin (*I am from…),* etc. Some said they would include that they were ill or were students at the school, often along with other information. In the interviews, some students could also describe developments in their introductions of themselves. One student introduced himself to the interviewer saying: *Hello, I am Paul, I am a musician*. After he had got a diagnosis a few years earlier he had introduced himself to a psychiatrist saying: *Hello, I am mentally ill, I am paranoid schizophrenic, and my name is Paul.*

Almost all students described significant changes in self-acceptance: *Because of my childhood and my background, I have always felt different and not good enough. I do not feel that way now.* They described being less shy or shameful and better able to accept their problems, but they also described feeling more secure and confident, and that they could manage, be alone, and cope: *I have it okay with myself. I can do a lot. That is a victory because I did not know that I could.*

### Personal engagement and integrity

Several students mentioned changes because of willpower. They described how they just “got up” and started making better choices and taking more responsibility in their lives again: *After a while, I realized that I had to do something myself.* When asked, they would say that this strength had always been a part of them, even if they had not used it for years. It was difficult for them to describe the process to “awaken” this willpower, but it seemed that time and acceptance, combined with beneficial relationships, was important in this process. The students also stated that they had gained more integrity and a stronger feeling of sense of self: *My ex*-*husband, he took control of my life, so I just disappeared. No one did that here. Here I am myself; I think for myself, do for myself.*

### Development

#### Changes in symptoms and strategies for coping

Most of the students had received psychiatric treatment before they attended the school, and some received different forms of treatment (e.g., medications or home visits from community nurses) while attending school. Two students had received psychotherapy earlier; both missed it and wished such therapy was available at the school.

Even though several students still received some treatment, most described a reduction in both symptoms and the need for treatment. Many talked about changes in symptoms and feeling less depressed, being more active, and having less anxiety; some said they had stopped using drugs or alcohol, or being self-destructive, and now had more stability or better concentration. One student described how, earlier, she stayed in bed all day, but now she was living an active, happy life: *Now I feel that I am “the old Anna” after a long period of sickness.*

Others described new strategies for coping; one man said he had started to keep a diary after being inspired by a lesson, and he felt this helped him a lot. Another described how, earlier, he used to be admitted to the hospital during the summer, but for the last two summers he had enjoyed painting outdoors, like the Skagen painters he learned about at school, and had not needed any admissions.

Most students said that, earlier, they had been admitted several times, some many times a year, but none reported any recent admissions. Several said they had been able to quit or reduce their medications with good results; it seemed that the need for treatment decreased when students had attended school for a while. Some students still needed support but typically less than they did earlier (e.g., sheltered living rather than long-term hospitalization, or medications but no acute admissions). Students also described being more actively involved in their treatment, and several mentioned that they had received support from their teachers in discussions with social services and therefore received better services than earlier.

The students seemed to represent two different groups. Some of the older students, mostly elderly persons with severe mental illness that had lasted over 15 years, described a positive development where they were now more stable. They said they no longer needed hospitalization, had more friends, lived a good life, were happier, had more control of their life, and, in general, were more satisfied with life, even if they still experienced symptoms, needed some treatment, and were unable to work. They described a close association between the conditions at the school and the changes they had experienced; they felt better when they attended school every week. They also described that, when the structure of school faded, for instance during the holidays, they would have a difficult time.

Others, mostly younger students, described that they were now able to study or work, had few or no symptoms, no longer took medications, and had no addiction problems any more. In addition, they also described personal developments; they said they had changed, and that the changes involved many parts of their lives. They described having more stability in life, that both they and others described them as better parents, and that it had become easier to keep appointments. They typically described these changes as stable and independent of attending school or not. Some students also described another development: in the beginning, they had a hard time with holidays, but now they could manage—not only without problems but also with joy.

### Personal growth, happiness, and hopes for the future

Most students described that they had regained their lives, had a better life, and had developed: *I see into myself and work with the difficult topics. That makes me a more integrated human. I develop. It is difficult to explain, but all the small, good experiences at school give my life so much quality, and then I can grow.*

They also described more-specific personal changes, such as having more stability and being more responsible; being more patient and less anxious; being able to speak in public; being a better friend or husband/wife/parent; being stronger; and having more energy. Some had started new hobbies, had gained a larger social network, or just felt more stable and confident: *I have a better life today. I have had many problems; it is as if my schooling helps me to enjoy my life better. I can manage my life now.*

Almost all students spontaneously stated that they are happier now and used words like “joy” and “happiness” to describe their lives—often remarking that this had changed a lot from their earlier experiences: *I have been very negative, but now I would say that I am very happy with my life, and I love to live, and I love all the things in life.* Many students also mentioned that they had acquired new knowledge, for example, IT knowledge, cooking, another language or other skills. This was important for them and gave them more opportunities in their everyday lives.

In the second interview, several students talked about changes since the first interview. Many had started working or studying at regular schools. Others had specific plans about education or work. For all of them, it was important that they changed and could combine continuing at school with new activities. For many students, it seemed to be important to help others in some way, for example, by getting an education in the social services or by giving talks for health care professionals. All students talked about plans and hopes for the future. Some plans were specific, such as getting an education or a job; others were more general, such as having a “good life”:

*Two years ago, I thought that I would die soon. I felt terrible; there was no future, and I could not see myself coping with anything. Now I am thinking I will have an education, I will have a normal life, a good job, a husband, education, a house and children. All these things many people take for granted. However, it is not obvious in my life. I am from a different world; for me it would be wonderful to achieve this. Now I think this is realistic to achieve. I did not think that before.*

## Discussion

In this section, similarities and differences between students and patients are discussed in relation to the research questions.

### How do patients and students describe their relationships and interactions?

Both students and patients described relationships as highly important. However, while students described in detail both qualitative and quantitative development in their relationships, patients mostly described an unchanging state of loneliness, short-term relationships or professional relationships, and little or no positive development regarding relationships.

There can be several ways to understand these differences. First, students and patients described clear differences in their *motivation* for relationships. Most students described their motivations for friendship as intrinsic and coherent. They talked about “having fun,” used cognitive concepts of “friends” and “families,” and weighed the mutual responsibility of friendship. Patients often mentioned external motivation for their relationships, such as wanting help from professionals or having the ability to learn from other patients’ experiences.

If the patient’s relationship to the therapist is “functioning” and the patient gets better, the relationship will be broken because the patient no longer needs it. However, he or she will still want the company of others, and this is especially important as patients may have few or no other relationships. Since these goals are contradictory, it might be problematic for them. Reaching one goal (“getting better”) will cause them to lose another goal (“having comforting company”), so maintaining secure relationships seems incompatible with getting better.

At the same time, they also described an intrinsic motivation for relationships—they enjoyed being with the nurses and other professionals, and talking and spending time with them. Of course, we cannot know if both these descriptions represent patients’ genuine experiences or if they simply replicate social norms. Ringer [19] has shown that patients often adapt their personal narrative to fit the narrative of the ward. This may be the case here, meaning that patients’ real motivations are intrinsic, but that they feel pressure to focus on the usefulness of the relationships.

Regardless of reasons, patients *did*, in fact, focus on this usefulness and did so in describing relationships with other patients—where the social norms of the wards might be less obvious. It is, therefore, possible that what has occurred is an adaptation of their concept of friendship, possibly affected partly by the social norms of the medical system. Some stated directly that therapists or nurses had told them that they should be with others to “work on their anxiety” or that “social relations might be good for their mental health” or for other external motivation. Although there may be truth to these, in regard to mental health, this might not be the best strategy for developing friendships. In a study of healthy adolescents, Ojanen et al. [20] found that adolescents who enjoyed the intrinsic qualities of friendship would develop more friendships, while those who established friendships based only on extrinsic motivation would lose interest over time. Even if their study is on adolescents, it might be relevant also for development for friendship in adult life. It could be harder for patients to develop friendships outside of professional relationships, since doing things “just for fun” indicating an intrinsic motivation was seldom described in the interviews.

Another striking difference between students and patients is the possibility to develop relationships over time. Students attended the schools much longer than patients remained at the wards and thus had the opportunity to develop lasting and mutual relationships. Most patients described relationships and situations that were fluctuating, and few had long-lasting relationships. Ojanen et al. [20] found that relationships and skills for building them need time to develop. If all relationships are brief, both skills for making lasting relationships and the motivation to do so may suffer. Most theories of development [6–8] state that consistent, long-term relationships are crucial for developing a secure and consistent sense of self. The students, however, described marked changes in social functioning and capacity for friendship.

Since impairments in social functioning and social cognition are common in patients with psychoses [21], many programs have been developed to correct cognitive biases [22]. The students had not received formal cognitive training but reported on development anyway. However, they might have benefitted from long-lasting and intensive social training in a natural environment. Individual Placement Support (IPS) is a method used for helping people with severe and long-term mental illness to function at work [23]. Instead of the traditional approach of training people first in a separate place, and then trying to transfer the learned skill to the workplace, IPS focuses on training people to do their jobs at an actual workplace, without the need to transfer skills, and this approach has shown better results than the traditional one [23]. Instead of training in cognitive skills separately, they get intensive and long-lasting practice, with teachers as role models, in real-life situations. This also aligns well with models of development stating that humans are natural social individuals and that all development occurs in social relationships [6–9].

### How do patients and students describe themselves, their motivations, and actions?

Both patients and students were receiving or had received psychiatric treatment; most stated that they had a diagnosis or had one earlier and that they were using or had used medication. However, issues such as treatment, medication, and illness had very different focuses in interviews with students and patients, and in their personal narratives; patients described themselves primarily as patients while students used mainly other narrative descriptions.

When asked about their everyday lives, students described a development where they enjoyed new activities and gained mastery, more relationships, and better experiences. They seldom used their illness to explain their actions or emotions (even if some stated that they used to do so earlier), and illness seemed to have little place in their lives and thoughts, suggesting that this was not their main narrative now. Instead, most of them used relational, social, or pedagogic explanations for their actions, emotions, and development, indicating that the schools’ focus on these themes had been internalized by the students.

Patients’ descriptions were quite different, with a much greater focus on their illnesses. In the interviews, both their descriptions of everyday life and their attributions and explanations of things that were happening were related to illness. They spoke of few topics other than illness, even when asked detailed questions about their lives. They also described little personal development or their development in the role of patient. Some mentioned that they wanted to do certain activities but had to wait until they were better; treatment of their illness had the highest priority.

Students, by contrast, did not wait. They described doing activities now; they had access to many more activities than did patients and mentioned these activities as important. It seemed that the different activities affected the students in several different ways. All students said that learning itself was important and, that it was crucial for them to feel that they could learn new things and experience mastery and growth. The students’ descriptions had a much greater focus on development, learning new things, increasing mastery, making changes, and personal growth. These are all central parts of the concept of flourishing and related to well-being and growth in humans [24]. Students also described that learning new skills gave them new opportunities. For example, if you can cook, you can invite a friend to dinner; if you know something about poetry, you can engage in conversation at a poesy festival. This aligns well with research findings that better social functioning and ability to engage in pleasant activities are connected to increased self-esteem and reduced depression in patients with severe mental illness [25].

The students also described that some of the theoretical and philosophical topics were important for them, and that reading about people in different life situations, for example, reading philosophy and history, gave them a broader view about their own lives and their problems. Dowrick et al. [26] found that attending reading groups and the content of the literature has significant effects on the outcomes of patients with depression [26], and reading groups have improved cognitive and psychosocial functioning of patients with psychosis [27]. This may indicate that not only participating in an activity but also the content of the activity might have affected the students.

Furthermore, not only the number of available activities but also motivation for activities differed between students and patients. Both students and patients talked about going for walks with others. Students did so in classes related to “nature” or “my community” or as part of art classes. The purpose of these walks was to learn about nature or their community/city or to paint. Descriptions of the walks were typically related to the purpose; they described the kinds of birds, flowers, or buildings they saw; the weather; what they learned; or what they painted. In addition, they often mentioned positive emotions and motivation, such as having fun or enjoying nature—all descriptions focusing on a motivation directly linked to the activity.

Patients described going for walks as part of treatment. Some stated that they enjoyed this, especially spontaneous trips alone with a nurse, when they could talk about their problems. They also went on walks together as a group, often framed as exercise. This seemed to be a less-popular activity, but the patients described that “exercise is good for treating mental disease.” The curative element, therefore, seemed to be the main purpose for these walks. Even when asked specific questions, they would seldom describe anything else about the trip (what they saw, the weather, etc.) but rather would repeat the extrinsic motivation of “treating mental disease.” Studies regarding the effects of physical exercise on severe mental illness [28] show that exercise, in fact, is beneficial, even if its effects on severe mental illness are less profound than for depression.

In this context, however, it is probably more important to consider the differences between the students’ descriptions of intrinsic motivation compared to the patients’ extrinsic motivation. Studies have repeatedly shown that extrinsic motivation is often less durable and weaker and might lead to lower creativity and engagement [29]. This might explain some of the differences between patients and students in regard to engagement. Students’ motivation seemed to be more intrinsic and internalized, and therefore easier to maintain over time. Patients, by contrast, often had a more extrinsic motivation (curing the disease), and this would be more difficult to maintain, especially when the reward (being cured) was not reached. Patients with schizophrenia tended to have an external locus of control and attributed their health to external factors associated with lower recovery [30]. These findings align well with the descriptions given by the interviewed patients but not with the students’ descriptions, indicating that the problem might lie in the social narrative surrounding the informants and not in their individual functioning.

Another difference between patients and students was the patients’ use of contradictory statements, which were not used by any student. There may be different interpretations of these phenomena. Ringer’s research [19] showed that patients often had to adapt their descriptions to align with the narrative of the ward, and that might be the case here—that patients say what they feel is expected of them, for instance, that they should work with their development, even if this does not align with reality. This interpretation fits well with the interesting shift in perspective in the statement quoted earlier, where patients start with a first-person perspective, *“I am working”* and then shift to a third-person perspective, *“one is working…,”,* indicating that this perhaps is not about him or her but about what he or she thinks is the expected thing to say. On the other hand, there are elements in the framing that could encourage this use of contradictions and especially lack of consistency. Most patients said that plans were often altered, and that there was little consistency between plans and reality. If the patients had learned to describe the world as it ought to be instead of how it is, it is natural that they would also use this pattern in their own descriptions.

Patients also tended to describe themselves in a passive and objectifying manner. Again, this may be a result of adapting to social norms at the ward, as Ringer described [19]. Ricoeur [31] explores the issue of self and identity, and states that all utterances must be interpreted in the context in which they appear, including who the person is talking to. When talking to a researcher about experiences with nurses, patients might felt a pressure to confirm to a norm indicating that others besides themselves know better what they need. They might also have been acclimated to a need to “prove” their statements by referring to professionals with greater authority then themselves. This means that the patients’ environment does not support and respect their personal autonomy and integrity, but rather encourages a process where others are experts about their lives. This is confirmed by many statements made by patients [15] indicating that, often, they felt disrespected by health professionals, or that their complaints or wishes were not taken seriously. Some of them also seem to have internalized this view, stating that the professionals “know better,” while others complained about not being taken seriously. Students, by contrast, did not describe themselves in an objectifying manner but instead as active agents. They used much of the interview time to describe their plans, wishes, dreams, choices, interests, and preferences. They stated that they were happy, had found themselves, had more energy, accepted themselves, were more confident, and lived better lives. They also said that they feel respected and accepted at school, and that the teachers take their statements, wishes, and needs very seriously.

The parental acceptance-rejection theory [32] states that there exists a general, cross-cultural connection between children’s perceived acceptance or rejection by their parents and their psychological adjustment. This theory aligns well with the descriptions of patients and students indicating that the difference in self-perception in the informants, as well as adjustment and happiness, are as least partly connected to how the students felt met by teachers/health professionals.

### How do patients and students describe their personal development?

There were clear differences between patients and students regarding descriptions of development. With few exceptions, most patients described stagnation or even worsening of their situation. By contrast, all students described some kind of development representing clinical recovery, personal recovery, or both [10].

Patients described receiving much more treatment than students did, especially repeated short-term interventions. These descriptions align well with research that finds sense of self to be a core challenge of schizophrenia [1, 2] and may also be relevant to other severe mental illnesses [3]. If the core problem for patients is related to sense of self, focusing on short-term interventions based mostly on symptoms and stabilization is likely to have limited long-term effects, just as the patients described.

The students’ diagnoses were similar to those of the patients, but they described much more development. They talked about less treatment but more stable and mutual relationships, respect, acceptance, and meaningful activities [15]. Again, these descriptions agree with the theories of sense of self as the core problem; thus, focusing on personal development might be more beneficial for reducing symptoms than treating symptoms directly.

Listening to the students’ descriptions, their experience of relationships, sense of self, and development seemed to interact in a constant process. This continuing development through long-term, mutual relationships and participation in meaningful activities closely resembles many of the classical theories for human development [6–9] describing the development of identity as a process of constant interaction and relationships that develops over time. This differs significantly from the patients’ descriptions of short-term interventions and aligns with the hypothesis of personal development as more crucial than treatment of symptoms.

A striking difference between students and patients was that patients talked much more about “usefulness” and treatment, while students described joy, fun, and liking their classmates. Many informants in both groups discussed problems starting in early childhood [15]. Several researchers state that early developmental trauma may negatively affect cognitive development and that play is important for repairing the impacts of trauma and re-establishing the conditions in which the brain can again learn and develop [33–35]. The constant focus on treatment and usefulness might reinforce the tendency in the traumatized brain to stay in what Siegel [34] describes as the “plane of activation.” A brain in this state will typically be locked into old solutions and negative thinking. To reach what he describes as the “plane of possibilities,” where the brain feels safe and able to playfully consider new possibilities, Siegel states that safety, time, and room for playfulness are necessary—the same features students mentioned as important for them.

There were also marked differences in the interviewees’ descriptions of hope. Patients in general described having little hope for the future, while all students talked about their hopes and plans for the future. Snyder’s Hope Theory [36] defines hope as bi-dimensional, combining agency and pathways. In regard to this theory, the patients’ lack of hopes and plans is natural, given the low agency and lack of pathways they describe. By contrast, the students’ self-acceptance, personal engagement, and experiences of mastery may facilitate their beliefs in further development in the future.

## Strengths and limitations

A strength of the study is the inclusion of persons (patients and students) from two different environments featuring different approaches and focuses

Since anomalies related to sense of self are more profound in psychoses than in personality disorders, including informants with different diagnoses might be problematic. With a small sample and no information collected from health services confirming diagnoses, we are not able to test whether there are any systematic differences between patients regarding diagnosis. But there were no obvious differences that seemed to be related to diagnostic groups or age, and both groups of informants reported ling duration of illness. Both patients and students told about several inpatient stays. But they were not asked about number or length of such stays, as this was not a main focus. Many told that they did not remember how many stays they had or the length of the stays, while others used phrases as “every summer” or “several times a year”. All information was given by the patients and students themselves, and no data collection was done from the health services in this qualitative study. The impression from the interviews is that both groups of informants had many inpatient stays, but quantification of this has not been a focus in this study.

The only systematic difference between the groups was nationality, and cultural differences might have affected the findings and interpretations. However, the Scandinavian countries are generally considered quite similar, which may limit any effect. The students’ descriptions support this view; most had received psychiatric treatment earlier, and their descriptions of treatment closely matched those of the Norwegian patients. It is therefore more likely that the differences described are associated with difference between being in treatment in mental health services and being in education, rather than different nationalities.

It could be argued that the patients, being inpatients at the time of the first interview, may have been in a more acute stage of illness than the students were. However, the inpatient units at the community mental health centers were not acute wards, but rehabilitation wards giving support to regain functioning for patients that seemed to be trapped in a chronic pattern of repeated smaller relapses. Both patients and students talked in a similar way about having had several hospital admissions. We found no indication that the differences between the two groups could be explained by differences in acuteness or severity of the illness.

As this is a qualitative study, no firm conclusions can be made regarding the causes of the differences described. Additional research, including quantitative research, is needed to confirm and further explore the suggested findings.

## Conclusions

Students and patients described several differences in how they experienced their relationships, themselves, and their development. Students described stable relationships and greater capacity for relationships; better quality of life; personal, social, and medical development; and intrinsic motivation for activities. Patients, by contrast, described a much higher focus on treatment, both by themselves and their health care providers. Paradoxically, most of the patients described only short-term or no positive development.

The findings and interpretations support research suggesting that anomalies in sense of self might be a core challenge for patients with severe mental disorders. For this group of patients, it is possible that their problems are closely related to identity development. Therefore, instead of rigidly focusing on treatment, it might be more effective to support patients’ personal and social development. Findings suggest that this could be done by offering intensive and lasting environmental conditions, including stable and mutual relationships, intrinsically motivated activities, and an environment that supports personal choices, acceptance, and development.

The study further suggests that the schools’ comprehensive and enduring aspects and daytime scheduling might be ideal for patients who need lasting and intensive care and those who do not benefit from long-term psychiatric inpatient and outpatient treatment. This group of patients has significant care requirements and needs more help than is currently available or offered. The study’s findings should be further explored to identify ways that these kinds of interventions might be relevant for patients’ long-term development. More research on use and adaptions of such environments offering treatment or education is needed to test this hypothesis of needs for different environments for different groups.
